# A stop-signal task for sheep: introduction and validation of a direct measure for the stop-signal reaction time

**DOI:** 10.1007/s10071-017-1085-7

**Published:** 2017-04-07

**Authors:** Franziska Knolle, Sebastian D. McBride, James E. Stewart, Rita P. Goncalves, A. Jennifer Morton

**Affiliations:** 1 Department of Physiology, Development and Neuroscience, University of Cambridge, Downing Street, Cambridge, CB2 3DY UK; 2 Institute of Biological, Environmental and Rural Sciences, Aberystwyth University, Penglais, Aberystwyth, Ceredigion SY23 4SD UK

**Keywords:** Two-choice discrimination, Behaviour, Go/no-go task, ADHD, Parkinson’s disease

## Abstract

Huntington’s disease (HD) patients show reduced flexibility in inhibiting an already-started response. This can be quantified by the stop-signal task. The aim of this study was to develop and validate a sheep version of the stop-signal task that would be suitable for monitoring the progression of cognitive decline in a transgenic sheep model of HD. Using a semi-automated operant system, sheep were trained to perform in a two-choice discrimination task. In 22% of the trials, a stop-signal was presented. Upon the stop-signal presentation, the sheep had to inhibit their already-started response. The stopping behaviour was captured using an accelerometer mounted on the back of the sheep. This set-up provided a direct read-out of the individual stop-signal reaction time (SSRT). We also estimated the SSRT using the conventional approach of subtracting the stop-signal delay (i.e., time after which the stop-signal is presented) from the ranked reaction time during a trial without a stop-signal. We found that all sheep could inhibit an already-started response in 91% of the stop-trials. The directly measured SSRT (0.974 ± 0.04 s) was not significantly different from the estimated SSRT (0.938 ± 0.04 s). The sheep version of the stop-signal task adds to the repertoire of tests suitable for investigating both cognitive dysfunction and efficacy of therapeutic agents in sheep models of neurodegenerative disease such as HD, as well as neurological conditions such as attention deficit hyperactivity disorder.

## Introduction

 Huntington’s disease (HD) is associated with profound changes in cognitive abilities (e.g., Stout et al. 2011; Papoutsi et al. 2014; Bates et al. 2015). Response inhibition is a crucial cognitive skill that allows successful and flexible interactions with a constantly changing environment, by interrupting an action that is no longer desired (Verbruggen and Logan 2008; Ridderinkhof et al. 2004). This cognitive ability is not only affected in neurodegenerative disorders such as HD (Rao et al. 2014; Wiecki et al. 2016), but also in Parkinson’s disease (e.g., Gauggel et al. 2004) and Alzheimer’s disease (e.g., Zancada-Menéndez et al. 2013), as well as in a variety of other psychiatric and psychological conditions such as schizophrenia (e.g., Enticott et al. 2008), obsessive–compulsive disorder (e.g., Menzies et al. 2007), hyperactivity (e.g., Solanto et al. 2001; Winstanley et al. 2006), impulsivity (e.g., Logan et al. 1997), alcoholism (e.g., Li et al. 2009; Noel et al. 2016), obesity (e.g., Nederkoorn et al. 2007), and gambling (e.g., Brevers et al. 2012; Lawrence et al. 2009).

Response inhibition requires the integrity of fronto-striatal circuitries (Eagle and Robbins 2003). A number of different tasks can be used to quantify response inhibition in rodents, including the five-choice serial reaction time task, the go/no-go task, and the stop-signal task (SST; see Eagle and Baunez 2010, for review). Each of these tasks monitors different aspects of response inhibition that are in turn thought to require different brain circuitry. The five-choice serial reaction time task, for example, monitors premature responding of a pre-activated response and is highly modulated by dopaminergic efferents into the ventral striatum (Christakou et al. 2004). The go/no-go task measures action restraint (i.e., preventing an action from starting), which is highly controlled by the inferior frontal cortex (Aron et al. 2004). The SST measures action cancellation (i.e., stopping an action that is already initiated) and is not only highly dependent on the inferior frontal cortex (Aron et al. 2004) but is even more reliant on normal functioning of the dorso-medial striatum (see Eagle et al. 2008, for review). Given that degeneration of the dorso-medial striatum is concomitant with early symptoms of HD (Vonsattel and DiFiglia 1998), the SST is a highly relevant task for monitoring HD progression (Rao et al. 2014).

We have developed a SST paradigm suitable for testing response inhibition in sheep. The sheep model for HD is particularly promising because not only are sheep useful models for studying HD (Morton and Howland 2013), but also HD sheep show HD-like pathology (accumulation of *HTT*-positive aggregates in the cortex with increasing age; Huntington’s Disease Sheep Collaborative Research Group et al. 2013), alterations in social behaviours (Morton et al. 2014) and changes in brain and liver metabolism (Handley et al. 2016, Skene et al. 2017) that resemble some of the changes found in HD patients.

**Fig. 1:**
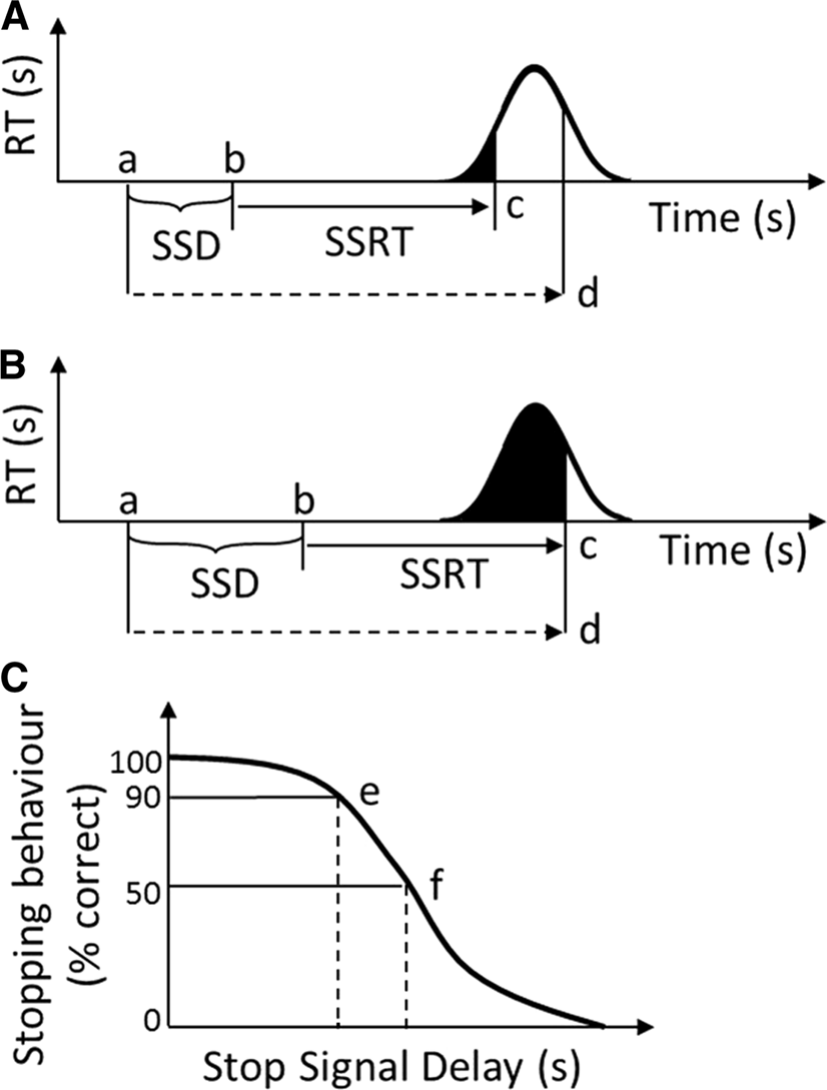
Assumptions and predictions of the race between the go- and the stop-process in the stop-signal task. The graphs in **a** and **b** show the race between the go- and the stop-process. The go-signal is presented at **a**, and the stop-signal is presented at **b**. The time between **a** and **b** comprises the pre-defined stop-signal delay (SSD, **b**–**a**). The go-process (*solid arrow*) finishes at **c**, whereas the stop-process (*dashed arrow*) finishes at **d**. Graph in **a** shows that with a short SSD the probability for a false response (*black area* under curve) is reduced and the probability for a correct inhibition (*white area*) is increased. Graph in **b** shows that with an increased SSD the probability for a false response (*black area*) is increased and the probability for a correct inhibition (*white area*) is reduced. The graph in **c** shows that with a short SSD (**e**), the probability for a correct stopping behaviour is higher than with a long SSD (**f**). SSRT (stop-signal reaction time) is the time required to successfully inhibit a response (Adapted from Eagle and Robbins 2003)

The critical measure of the SST is the stop-signal reaction time (SSRT), which is the time required to respond to the presentation of the stop-signal. Thus, the SSRT measures the time to inhibit a pre-activated response (Logan and Cowan 1984; Logan et al. 2014). Theoretically, the latency of the SSRT is driven by a race between a go-process that is initiated to cause the response and a stop-process that interrupts the already-initiated response (Fig. 1). A response is only successfully stopped if the stop-process finishes prior to the go-process (Logan et al. 2014).

In this study, we report the development and validation of a novel SST paradigm suitable for use in sheep and other large animals, such as pigs or cattle. In the conventional SST paradigm used for humans, rodents and monkeys, a finger or paw movement is required in response to a stop-signal (Logan and Cowan 1984). The SSRT is measured indirectly by subtracting the stop-signal delay (i.e., time difference between the stop and the go-signal, SSD) from a value of the time necessary to complete a go-response (go-RT). This specific go-RT is derived from the distribution of go-RTs in relation to the probability of responding incorrectly to the stop-signal (explained in more detail in the Methods). An increased SSD reduces the probability of a successful stop, whereas a decreased SSD enhances the probability for a successful stop (Fig. 1; Middelbrooks and Schall 2014). In contrast to the conventional approach of estimating the SSRT, we measure the SSRT directly by using a technique which captures the complete course of movement of quadrupeds, such as sheep. Our results show that sheep are able to acquire a complex cognitive task that incorporates both response inhibition and a revision of this response. The SST paradigm for sheep can be used to measure response inhibition deficits in sheep models of neurodegenerative disorders, such as HD, as well as other neurological and psychiatric disorders for which sheep models might be developed.

## Materials and methods

### Animals

We used nine sheep (Welsh Mountain, all females, aged 7–8 years, 45–70 kg) that were permanently held in a flock at the University of Cambridge. All sheep lived outdoors with free access to grazing, water, and shelter. During training and testing in this experiment, the sheep received a food supplement of no more than 200 g cereal-based pellets each day (Badminton Country Sheep Nuts, Badminton Country Feeds, UK). The pellets were used as the reward throughout the study. All sheep had previously been used for cognitive testing (McBride et al. 2014; Morton and Avanzo 2011). The sheep’s weights were recorded every 3 weeks. The study was carried out in accordance with the UK Animals (Scientific Procedures) Act, 1986 and did not require the use of any regulated procedures. No further ethical approval was required.

### Equipment and technologies used for the stop-signal paradigm

**Fig. 2:**
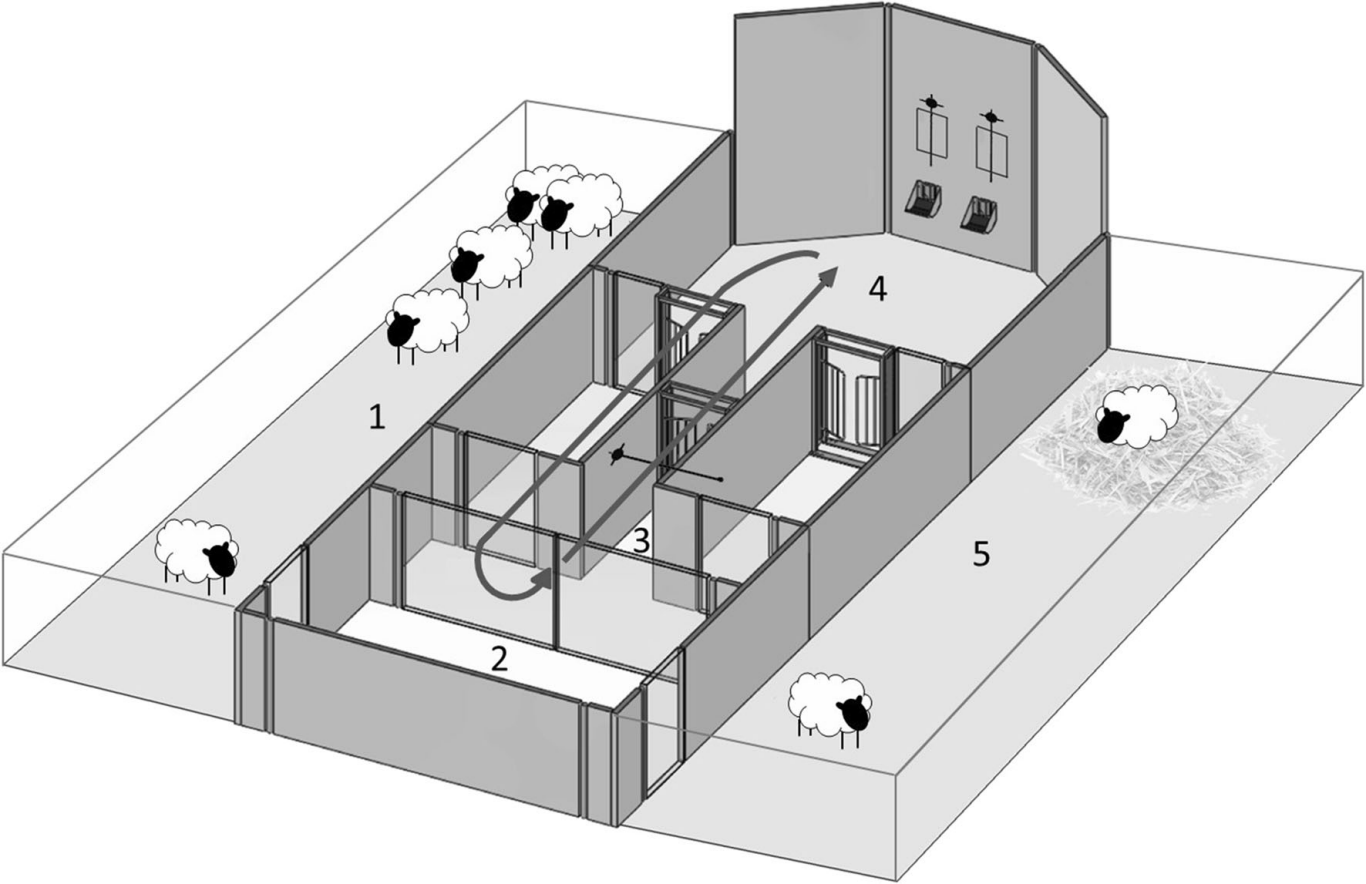
Operant system set-up for stop-signal task. All sheep stay in the waiting pen (*1*) until brought separately into the pretesting area (*2*). They enter the one-way ambulatory circuit (*solid arrow*) via the entry corridor (*3*). The sheep self-activate each trial by passing an infrared sensor () in the corridor that leads to the testing area (*4*). The visual stimuli are presented on two screens () in the testing area. An infrared sensor above each screen captures the selection behaviour of the animal. Activation of the sensor either initiates the dispensing of a food reward into the feed trough () or generates an error signal. After receiving a reward (or error signal), the sheep proceeds through a one-way gate () to the beginning of the one-way ambulatory circuit. After the session is completed, the sheep is taken back into the pretesting area (*2*). From there the sheep is released into the resting pen (*5*) (Adapted from McBride et al. 2016)

**Fig. 3:**
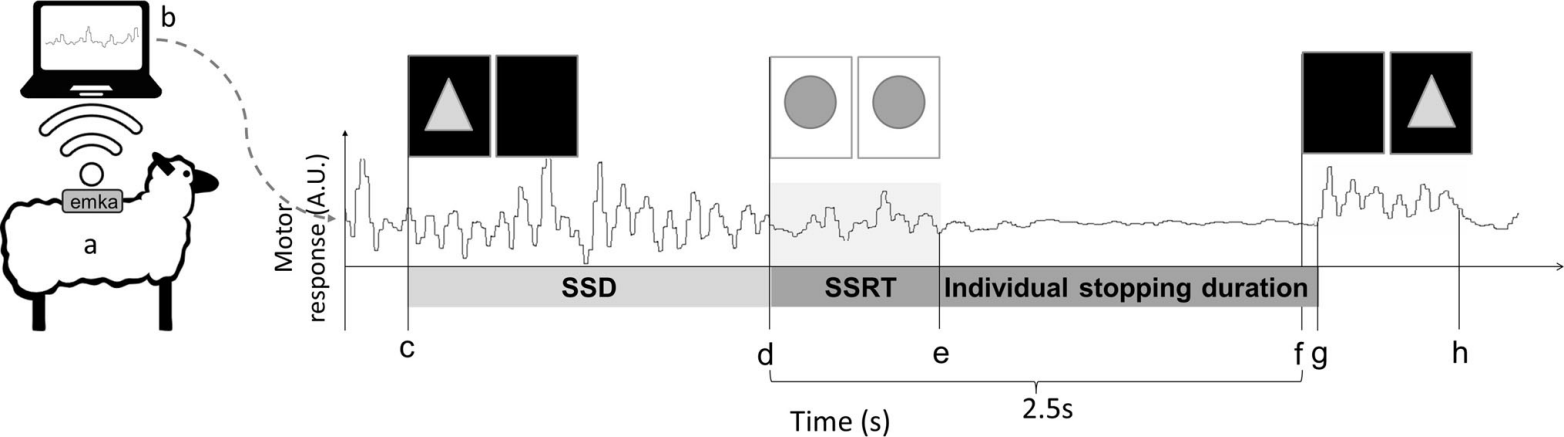
Cartoon of the system set-up for the stop-signal task showing a typical response from the accelerometer during the experiment. The emka telemetry pack is attached on the back of the sheep (*a*). When the sheep moves, a motor response is generated. This accelerometer signal is captured from the telemetry pack and wirelessly transmitted to a PC (*b*). Upon activation of the trial (*c*), the go-signal is presented on one of two screens. The stop-signal is presented on both screens (*d*) after a pre-defined stop-signal delay (SSD,* d*–*c*). The time between the presentation of the stop-signal and the amplitude drop of the motion track at e in this example represents the stop-signal reaction time (SSRT). The stop-signal is presented for 2.5 s. Thereafter the go-signal reappears at *f*. The sheep starts to walk (*g*) and makes a choice at the screens (*h*). The sheep is rewarded for selecting the screen presenting the triangle. *SSD* stop-signal delay, *SSRT* stop-signal reaction time, stop-signal presentation time = 2.5 s

 The study was conducted using a semi-automated operant system placed in an outside pen area (Fig. 2; McBride et al. 2016). This comprised two holding pens and a testing pen. The testing pen contained an ambulatory circuit, two screens for stimuli display, and food dispensers. During the task, the sheep moved through the circuit triggering the sensors placed above the screens that presented the signals. In order to monitor the movement of the sheep and to capture the SSRT, we used a back-mounted telemetry accelerometer (emkaPACK, telemetry system, emka Technologies S.A., Paris, France). The signal of the emka telemetry system was transmitted wirelessly to a computer (Fig. 3), where it was recorded using a system-specific acquisition software, iox2 (version 2.5 rev. 3, 2010, emka Technologies S.A., Paris, France). Iox2 was also used to analyse the motion tracks. We used MATLAB R2013a (MathWorks, Natick, MA, USA) in combination with Psychtoolbox (PTB-3, psychtoolbox.org) to programme all parts of the experiment as well as to capture the behavioural data. Input from sensors and output to feeders as well as the telemetry system was transferred to MATLAB and recorded in an output file, using a 12-bit USB data acquisition device (USB-1208 fs, Measurement Computing, Norton, MA, USA).

### Testing procedure

All sheep performed one or two 10–15 min sessions per day. At the beginning of each session, all sheep were brought into a waiting pen, where they had free access to water. Sheep were tested singly. For training and testing, sheep entered the testing pen of the operant system in no defined order. During all sessions of the training and the testing stage, each sheep self-activated each trial by passing an infrared starting sensor placed in the entering corridor. The visual stimuli were presented on two screens about 3 m from the starting sensor. Auditory stimuli were presented over speakers placed beside the screens. An infrared sensor above each screen captured the selection behaviour of the animal and initiates the dispensing of a food reward or an error signal. In order to activate the sensor, the sheep had simply to break the light beam. This could be done by the animal putting its head into the food trough or by exploring the screen. After the final trial, the sheep exited the testing area into the pretesting area. After completion of the Training Stage 3 and the testing stage, the accelerometer was detached from the sheep’s back and used for the next sheep. The sheep was then released into the resting pen where it had free access to water and straw (Fig. 2). After all sheep had completed a session, they were released to the field.

All sessions were video-recorded to observe the behaviour of the animals and possible disturbing factors (e.g., noise) during the experiments. It was also useful and interesting for other members of the team to watch the behaviour of the animals. This would not be possible without video recordings, since only a single experimenter was present during the training and testing. Although no quantitative analyses were conducted in the current project, this would be possible retrospectively.

During all phases, the experimenter maintained a passive posture in the pretesting area, avoiding sudden movements and not interfering in the task. This allowed the animals to perform the task at their desired pace.

### Stop-signal paradigm for sheep: training and stop-signal testing

#### Training

**Fig. 4:**
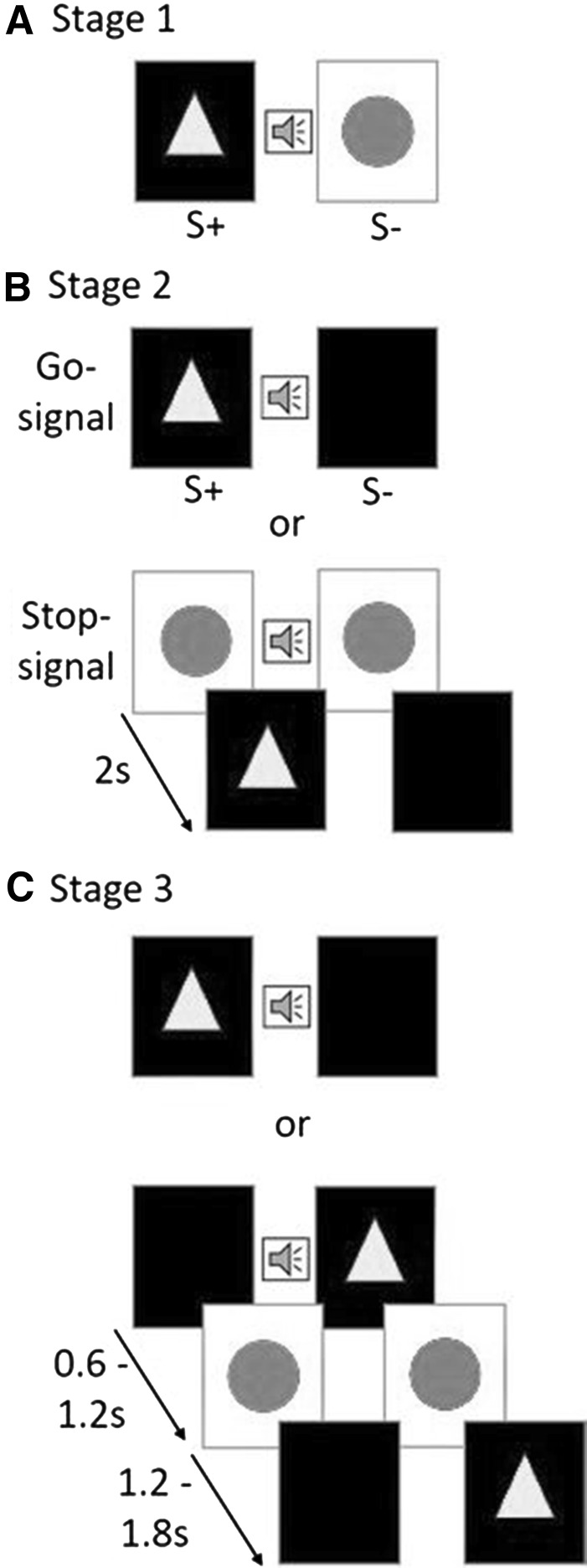
Presentation of stimuli during the three stages of training. In **a** (Stage 1), a two-choice discrimination set-up is shown, which consists of S+ (*yellow triangle* on *black* background) and S− (*blue circle* on *white* background). The visual stimuli are presented in conjunction with an audible sound (). In **b** (Stage 2), one of two sets of visual stimuli is presented in each trial in conjunction with an audible sound. The stimuli are either the go-signal, or a set of visual stimuli comprising the stop-signal (consisting of a blue circle on both screens), followed after 2 s by the go-signal. In **c** (Stage 3), one of two sets of stimuli is presented per trial in conjunction with an audible sound. Either the go-signal or a set of visual stimuli comprising of a go-signal, a stop-signal, and a go-signal is presented. In Stage 3, the first go-signal is presented with a pre-defined delay (stop-signal delay) of 0.6–1.2 s; the stop-signal is presented for 1.2–1.8 s (colour figure online)

 In each training stage, sheep were familiarized to a new aspect of the SST testing (Fig. 4a). The sheep moved onto the next training stages once the learning criterion was met. The criterion was set to 6 consecutive correct responses (*P* = 0.0015) or 80% correct (*P* = 0.0012).

#### Training Stage 1

 Training Stage 1 consisted of a two-choice discrimination task. For each trial, two different visual stimuli (S+, S−) were presented on two screens in conjunction with an audible sound (750 Hz, 500 ms). The S+ was a yellow triangle on a black background, and the S− was a blue circle on a white background. Side allocation of S+ and S− was pseudorandomized. The selection of S+ resulted in a food reward (5 g cereal-based pellets), whereas a selection of the S− resulted in the presentation of a high-pitched error sound (1000 Hz, 500 ms) and both screens turning black. Each trial was time-limited to 15 s after activation of the activation sensor. Timeouts were indicated with a short high-pitched sound (2250 Hz, 300 ms). Correction trials were implemented in this training stage, whereby an incorrect choice or timeout resulted in the same trial being repeated until the sheep made the correct choice. A low-pitched sound (260 Hz, 1900 ms) indicated the end of the session. Each session consisted of 10 trials. All sheep completed a minimum of 8 and a maximum of 10 sessions. Stage 1 familiarized the sheep with the specific S+ and S− stimuli as well as exposing them to the two error types (i.e., choice error, timeout) that were used throughout all training stages and the final experiment.

#### Training Stage 2

Training Stage 2 familiarized the sheep with the stop-signal, and the concept of waiting until the go-signal appeared. Training Stage 2 consisted of two different sets of visual stimuli that were presented at equal probability. Upon initiation, visual stimuli were presented in conjunction with an auditory stimulus (750 Hz, 500 ms). The first set of stimuli comprised a two-choice discrimination consisting of the same S+ (yellow triangle on black background) that was used in Training Stage 1, but with a black screen as the S− on the second screen. At initiation, the visual stimuli were presented in conjunction with an auditory stimulus (750 Hz, 500 ms). The sheep was rewarded for choosing S+, and was presented with the error signal used in Stage 1 when an incorrect choice was made (i.e., selection of S−). The yellow triangle on a black background now constituted the go-signal. The second set of stimuli consisted of two stepwise presentations of visual stimuli. In the first step, a blue circle on a white background (previously the S−) was presented on both screens simultaneously in conjunction with an auditory stimulus (750 Hz, 500 ms). This double presentation of the S− constituted the stop-signal. The stop-signal was presented for 2 s. During the presentation of the stop-signal, the feeding sensors could not be activated. The animal was required to wait until the go-signal appeared and was rewarded for selecting S+. If the animal approached the screen while the stop-signal was presented, no error signal was provided. The second step represented the ‘go signal’ (yellow triangle/S+, black screen/S−). Stage 2 also contained correction trials, and each trial had a ‘timeout’ of 15 s. Timeouts were indicated with a short high-pitched sound (2250 Hz, 300 ms). The end of session was again indicated using the low-pitched sound (260 Hz, 1900 ms). Each training session contained 10 trials. Each sheep completed 10 sessions.

#### Training Stage 3

In Training Stage 3, the sheep were familiarized with the concept of interrupting an already-started movement. Training Stage 3 consisted of two different sets of visual stimuli presented at equal probability (50%). At initiation, each set of visual stimuli was presented in conjunction with an auditory stimulus (750 Hz, 500 ms). The first set of stimuli presented the go-signal used in Stage 2 (yellow triangle/S+, black screen/S−). Again the sheep were rewarded for selecting the S+. The second set of stimuli contained three stepwise presentations of different stimuli. The first step was the go-signal (yellow triangle/S+, black screen/S−), the second step was the stop-signal (blue circle on both screens), and the third step was the representation of the go-signal with the yellow triangle (S+) appearing on the same screen as the first presentation. During Training Stage 3, the SSD, the delay between the first go-signal and the stop-signal, was gradually increased from 0.6 to 1.2 s (Table 1). A prolonged SSD forced the sheep to approach nearer to the screen before the stop-signal was presented. The duration of the presentation of the stop-signal was also increased gradually in a pre-defined manner from 1.2 to 1.8 s (Table 1), to help reinforce the sheep’s stopping action and fully stop the movement. At this stage, an error signal was also introduced whereby activation of the feeding sensors during the stop-signal resulted in an error signal (1000 Hz, 500 ms). Stage 3 did not include correction trials. Each trial was time-limited to 15 s after activation of the start sensor. Timeouts and end of session were indicated using a short high-pitched sound (2250 Hz, 300 ms) and a low pitch sound (260 Hz, 1900 ms). A training session contained 14 trials. In total, 35 sessions were conducted during this training stage.

**Table 1:** Stop-signal delay and stop-signal presentation time over 35 sessions during Training Stage 3

Session	Stop-signal delay (s)	Stop-signal presentation time (s)
1–5	0.6	1.2
5–10	0.6	1.4
11–15	0.6	1.6
16–20	0.8	1.6
21–25	0.8	1.8
26–30	1.0	1.8
31–35	1.2	1.8

During Training Stage 3, sheep were also familiarized with the procedure of surcingle and accelerometer attachment. The telemetry accelerometer (described above) was used to capture the motion and stopping behaviour of each sheep. This procedure did not limit their ability to move or perform their normal behaviour.

#### Testing stage of the stop-signal task

The testing stage involved the presentation of two sets of stimuli that were also used in Training Stage 3. The go-signal displayed a yellow triangle (S+) on one screen versus a black screen (S−), which was presented in conjunction with a sound (750 Hz, 500 ms). The stop-signal was a blue circle on a white background presented on both screens. Go-trials consisted only of the presentation of the go-signal. Stop-trials consisted of the stepwise presentation of a go-, a stop-, and a go-signal as described in Training Stage 3. In a stop-trial, the stop-signal was presented with an individual animal-specific delay, the SSD. This specific SSD was calculated as one-third of the individual mean reaction time in response to the go-signal (go-RT) of trial 2–4 (latency between initiation of the trial and the screen sensor). We chose one-third empirically. After this delay, the sheep were about 1 m away from the screen, which was the minimum distance needed for them to perform a successful stop. During the first four trials, no stop-trials were implemented. The go-RT of the first trial was omitted because the sheep usually entered the testing area with a pace faster than their average walking pace throughout the experiment. The mean go-RT of trials 2–4 did not significantly differ from the mean of any other three go-RTs randomly chosen from the same session. This animal-specific SSD accounted for the individual latencies between initiation of a trial and reaching the screens, which was determined by the animal’s walking speed.

After the individual sheep-specific SSD, the stop-signal was presented for 2.5 s (stop-signal presentation time). The sheep was required to interrupt its movement completely, rather than just slowing down its walking pace. The sheep was required to inhibit its response for the length of the presentation of the stop-signal in order to perform a successful stop. After the time for the presentation of the stop-signal was up, the go-signal (S+) reappeared randomly on either screen.

Go-trials were presented in 78% of the total number of trials and stop-trials in 22%. In both trial types, the sheep received a food reward for selecting S+. Error signals were presented for either the selection of a wrong stimulus (S−) or for the activation of the feeding sensor, while the stop-signal was being presented. Timeouts occurred when the sheep did not activate either of the two feeding sensors within 15 s of initiating the start sensor. The end of the session was marked by a prolonged low-pitched sound. The SST testing consisted of 18 trials including four stop-trials, with no correction trials, and 25 sessions. Thus, in total all sheep competed 450 trials including 100 stop-trials. Before each session, the surcingle and accelerometer was attached to the back of the sheep.

### Data acquisition and analysis

#### Conventional approach: estimation of stop-signal reaction time

We estimated the SSRT using the standard procedure used in other studies (e.g., Logan and Cowan 1984; Eagle et al. 2008). For each of the last five sessions, we generated a ranking of the go-RTs. We then took the *n*th reaction time, which was calculated by multiplying the probability of performing an incorrect stop with the total number of go-RTs in that particular session. The SSRT was estimated by subtracting the corresponding SSD from the *n*th go-RT. For example, during a session of 162 trials, including 36 stop-trials and 126 go-RTs, go-RTs were ranked in order of lowest to highest duration. During this session, correct stops were performed with a probability of 0.90 and, accordingly, the probability of failed stops was 0.1. The probability of failed stops was then multiplied by the total number of go-RTs (126) to generate *n* which was 13. We then took the 13th go-RT from the ranked list, to give an estimated go-RT value of 1.5 s. The corresponding SSD was 0.60 s. We subtracted the SSD from the 13th go-RT to give an SSRT value of 0.90 s.

#### Novel approach: direct measurement of the stop-signal reaction time

**Fig. 5:**
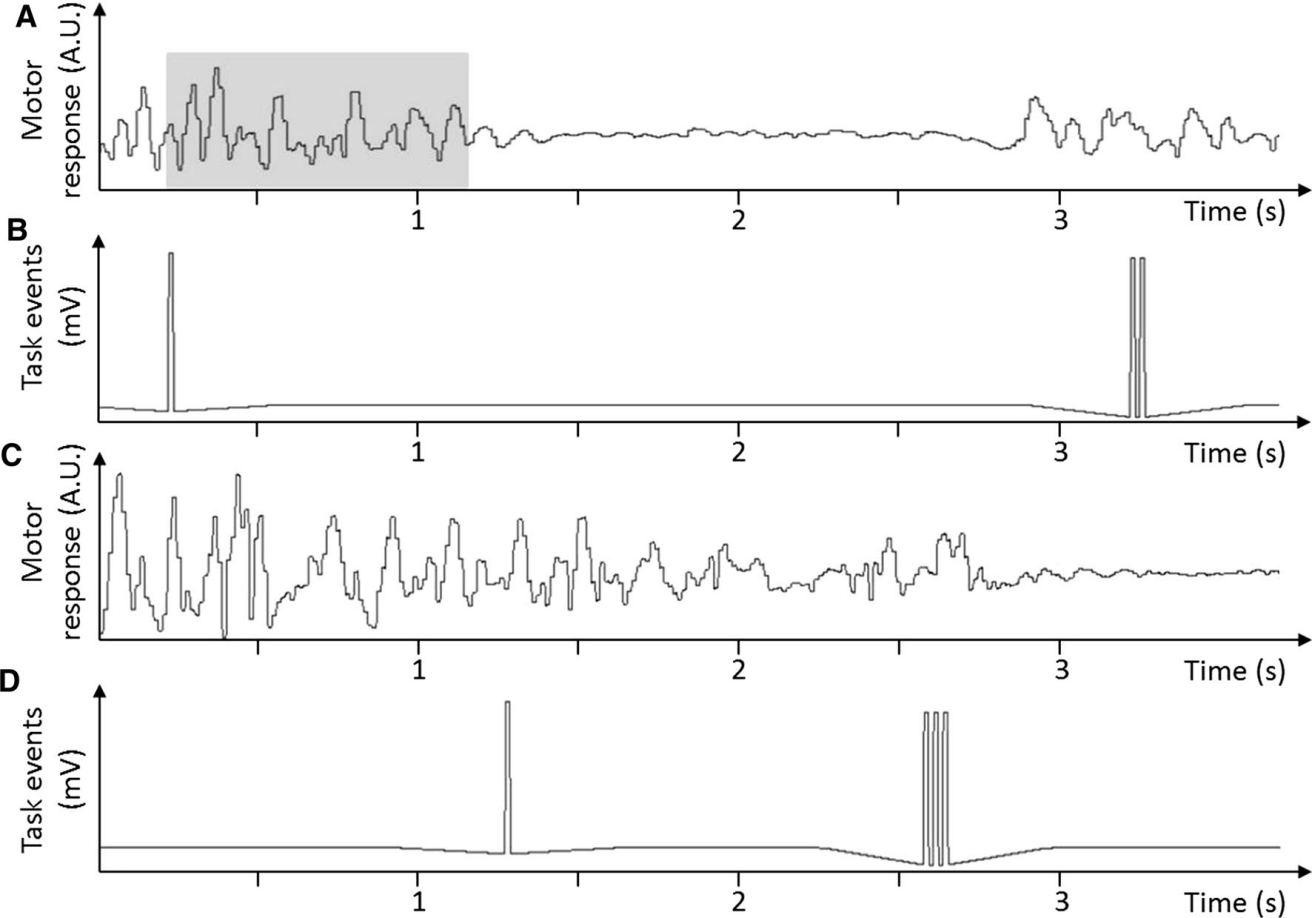
Capturing of movement and main events of the stop-signal task. In **a**, the motion track of correct stopping behaviour is shown. The corresponding main events of the stop-signal task (SST) are shown in **b**. The first 5 mV peak in **b** symbolizes the onset of the presentation of the stop-signal. The two 5 mV peaks at ~3.25 s show the reward presentation for making a correct decision after inhibiting a response to the stop-signal. *Shaded area* in **a** represents the stop-signal reaction time (SSRT), which is the time from the presentation of the stop-signal until the sheep stops. In **c**, the motion track for incorrect stopping behaviour is shown. The corresponding main events of the SST are shown in **d**. The first 5 mV peak in **d** symbolizes the onset of the presentation of the stop-signal. The three peaks at ~2.6 s represent the error signal provided for an incorrect respond to the stop-signal

We used the back-mounted telemetry system to capture the individual SSRTs directly. During a correct stopping behaviour, the sheep fully interrupted its movement for the presentation time of the stop-signal. The emka capturing tool iox2 made the stopping behaviour visible on a motion track (Fig. 5). Interfaced with the MATLAB code, the motion track also included all main events as trigger point, such as the presentation of stimuli and activation of feeders. After presentation of a stop-signal, the onset of the stopping behaviour was detected if the amplitude of the emka slow waves dropped by 500 mV over 100 ms. Thus, the SSRT was directly measured as the timing between the presentation of the stop-signal stimulus and the beginning of the stopping behaviour (Fig. 5).

#### Statistics

We measured SSRTs, go-RTs, and correct stopping behaviour (i.e., a correct response to a stop-signal). All statistical analyses were conducted and figures were drawn using GraphPad PRISM 5.04 (GraphPad Software, Inc., 7825 Fay Avenue, Suite 230, La Jolla, CA 92037 USA). Mean ± SEM is presented for all data. We used unpaired Student’s t tests, one-way analysis of variance (ANOVA) with Newman Keuls post hoc tests, where applicable, or Pearson’s correlation. For all measures, we compared performance during the first five sessions with performances during the last five sessions. The first five sessions were representative of their initial performance, whereas the last five sessions represent the maximum performance. The threshold for statistical significance was set at *P* ≤ 0.05.

### Results

#### Training

All animals successfully completed all training stages. Training Stage 1 was set up to introduce the two-choice discrimination task. By the tenth training session, all sheep had reached the learning criterion of 80% correct. In Training Stage 2, the sheep learned to wait until the two-choice set of stimuli was presented on the screen. After two sessions, all sheep had reached the learning criterion on the longest presentation of the stop-signal. During Training Stage 3, the response inhibition was introduced using a short presentation of the stop-signal. Comparing the results of Sessions 1–5 to Sessions 31–35, the sheep significantly improved their correct response to a stop-signal, from 6.8% ± 0.02 to 51.93% ± 0.06. They significantly improved their performance in the two-choice discrimination from 71.18% ± 0.03 in Sessions 1–5 to 87.0% ± 0.02 in Sessions 31–35.

#### Stop-signal testing

##### Stopping and choice performance

In the SST testing, all sheep completed 25 sessions, with a total of 450 trials including 100 stop-trials. We analysed the choice performance during no-stop-trials and stop-trials, correct stopping behaviour, individual SSRTs for correct stops and go-RTs. Although the sheep improved their stopping behaviour over the course of the experiment, they were already significantly above chance in Session 1–5 (*P* < 0.001, *t* = 9.37, *df* = 4). In Sessions 1–5, they performed a successful stop in significantly fewer cases (75.55 ± 2.73%) compared to Sessions 21–25 (90.71 ± 1.02%) of the experiment (*P* < 0.001, *t* = 5.20, *df* = 8). The choice behaviour after a successful stop in Sessions 1–5 (92.22 ± 3.41%) was not different to that of Sessions 21–25 (94.14 ± 2.99%) of the experiment (*P* = 0.68, *t* = 0.42, *df* = 8, *F* = 1.30). This performance did also not differ significantly (*P* = 0.46, *r* ^*2*^ = 0.15, *df* = 19, *F* = 0.91) from the choice behaviour in the go-trials, where they performed to an equally high level (*P* = 0.56, *t* = 0.61, *df* = 8, *F* = 1.45) during Sessions 1–5 (97.06 ± 0.80%) and Sessions 21–25 (96.42 ± 0.66%).

##### Measurement of stop-signal reaction time

**Fig. 6:**
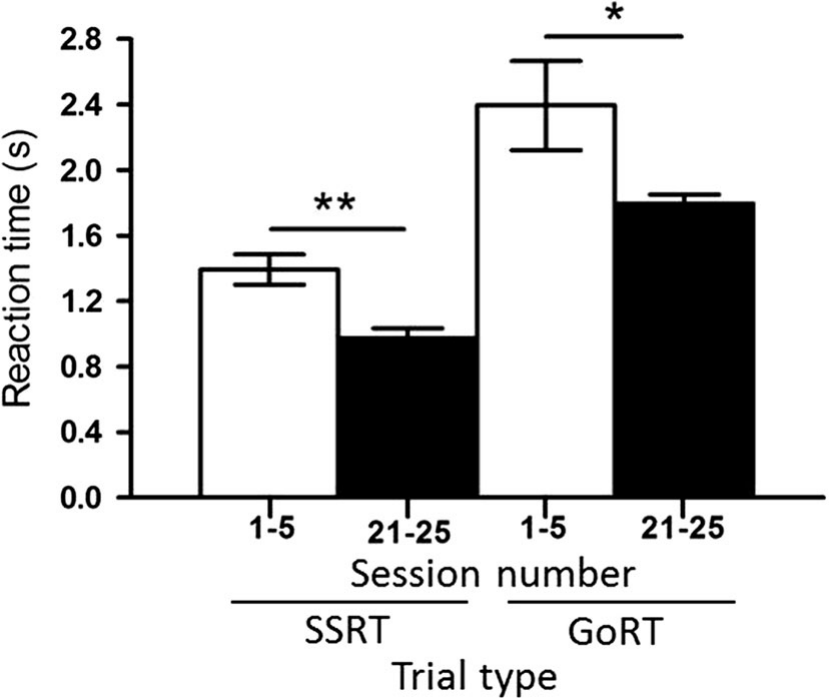
Effects of training on average reaction times during stop- and go-trials. Data show group means (±SEM) of SSRT and of go-RT during Sessions 1–5 (open columns) and Sessions 21–25 (closed columns). *SSRT* stop-signal reaction time, *go-RT* go reaction time; *P < 0.05; **P < 0.001

**Fig. 7:**
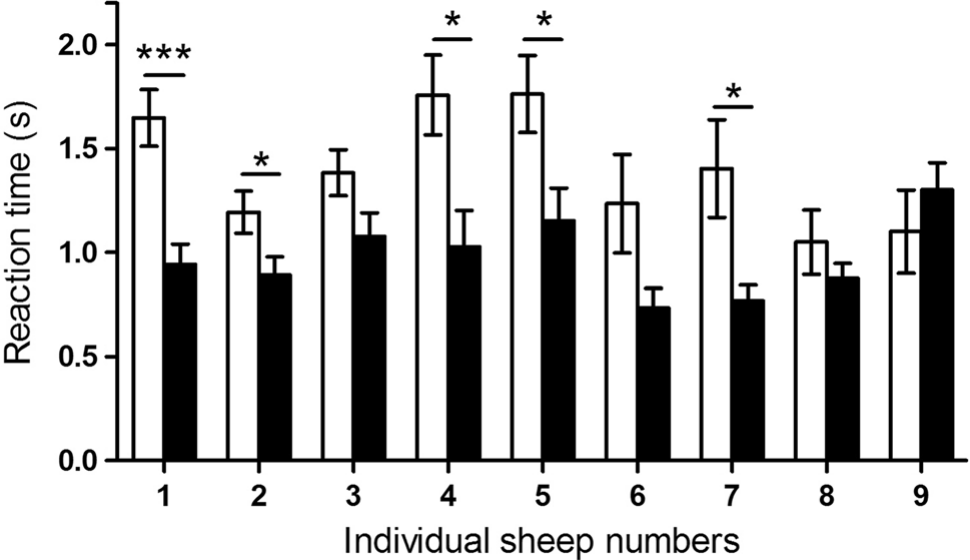
Effects of training on stop-signal reaction times of individual sheep. Mean SSRT (±SEM) are compared between Sessions 1–5 (*open columns*) and Sessions 21–25 (*closed columns*). *P < 0.05; ***P < 0.0001

Using our novel approach to measure the SSRT directly, we found that over the course of the experiment sheep were able to respond significantly faster to the stop-signal in Sessions 21–25 compared to Sessions 1–5. The group average of the SSRT was significantly reduced (*P* < 0.001, *t* = 5.20, *df* = 8) from 1.393 ± 0.06 s in Sessions 1–5 to 0.974 ± 0.04 s in Sessions 21–25 (Fig. 6). The individual SSRTs (Fig. 7) show that five of the nine sheep decreased their SSRT significantly when comparing the beginning and the end of the experiment (Table 2). Using the conventional approach, the average SSRT Sessions 21–25 were 1.058 ± 0.03 s. This was not significantly (*P* = 0.17, *t* = 1.39, *df* = 113, *F* = 2.90) different from the directly measured SSRT of 0.974 ± 0.04 s. Similarly, the go-RT decreased significantly (*P* = 0.03, *t* = 2.12, *df* = 86) from Sessions 1–5 (2.395 ± 0.27 s) to Sessions 21–25 (1.796 ± 0.05 s). Additionally, the variance was significantly (*P* < 0.001, *F* = 26.14, *DFn* = 44, *Dfd* = 42) reduced (Fig. 6).

**Table 2:** Individual SSRTs for nine sheep comparing the first to the last five testing sessions

Sheep	Mean time (s) ± SEM		*P*	*t*, *df*
	Sessions 1–5	Sessions 21–25		
1	1.65 ± 0.14	0.94 ± 0.1	<0.001	4.203, 14
2	1.19 ± 0.1	0.89 ± 0.09	<0.05	2.268, 14
3	1.38 ± 0.11	1.08 ± 0.11	n.s.	1.940, 14
4	1.76 ± 0.19	1.03 ± 0.18	<0.05	2.804, 14
5	1.76 ± 0.19	1.15 ± 0.16	<0.05	2.511, 14
6	1.24 ± 0.24	0.73 ± 0.09	n.s.	1.965, 14
7	1.40 ± 0.24	0.77 ± 0.08	<0.05	2.568, 14
8	1.05 ± 0.15	0.88 ± 0.07	n.s.	1.026, 14
9	1.10 ± 0.20	1.2 ± 0.13	n.s.	0.846, 14

*n.s.* not significant

**Fig. 8:**
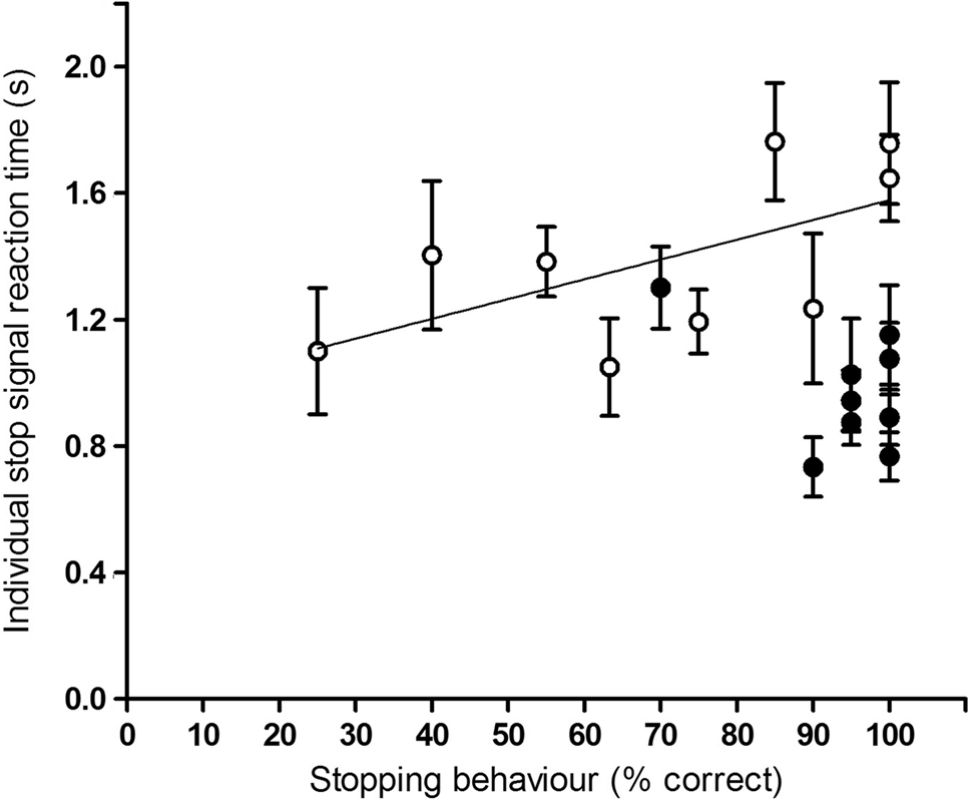
Relationship between the stopping behaviour and the SSRT of individual sheep. Data show the mean SSRT (±SEM) of individual sheep (*n* = 9) plotted against the corresponding stopping behaviour. During Sessions 1–5 (*open circles*), the correlation shows a trend towards significance [*r*(9) = 0.605, *P* = 0.08, *r* ^*2*^ = 0.001]. No correlation was found during Sessions 21–25 (*closed circles*)

In order to investigate whether the number of correct stops was influenced by the length of the SSRT, we correlated individual SSRT values and the performance during the stop-trials (i.e., percentage of correct stops; Fig. 8) within Sessions 1–5 and Sessions 21–25. We also correlated group average values of SSTR and performance during the stop-trials over the total of 25 sessions. No significant correlations were observed although there was a trend to significance [*r*(9) = 0.605, *P* = 0.08, *r* ^*2*^ = 0.01] in Sessions 1–5 of the final SST experiment.

We correlated individual SSRT with the body weights of the sheep to investigate the impact of inertia on the stopping behaviour. No significant correlation was found.

## Discussion

Here we describe the design and validation of an SST paradigm for use in sheep. Our results show that after 25 sessions the sheep learned to respond correctly to the stop-signal in ~91% of trials. The sheep performed equally well on the discrimination task during the go-trial and in the stop-trial. Our findings show that sheep are able to inhibit an already-started response with a reaction time of about 1 s and successfully revised their selection behaviour after the stop-signal. The validated SST for sheep described here, therefore, has the potential to monitor the progression of cognitive decline in HD sheep.

We used a back-mounted accelerometer to directly measure the SSRT. This approach has not been used in any other study. The telemetry system captures the whole sequence of a movement and provides a direct read-out for the SSRT. By contrast, the conventional SST set-up provides an estimated measure of the SSRT only (see, Eagle et al. 2008; Logan and Cowan 1984; Logan et al. 2014), as described in the Methods. Both the directly measured and the estimated SSRT approach gave similar values for our study, which confirmed the validity of the capturing tool. The direct measurement approach is especially useful for large animals such as sheep, where training of simple sequences of movements (e.g., using only one fore limb) would require long training periods. Furthermore, the directly measured SSRT is independent of other measures such as the go-RT and probability of correct stops and significantly fewer trials are necessary to obtain a reliable SSRT measurement. The direct accelerometer-based technique could also be applicable for rodents, which would allow the measurement of SSRT for a greater range of behaviours, from simple to complex sequences, with a reduced number of trials.

Our data showed that the SSRT of sheep (~1 s) is about five to eight times slower than to other species (humans: 200–480 ms, e.g., Logan and Cowan 1984; Middelbrooks and Schall 2014; rats: 120–280 ms, e.g., Feola, et al. 2000; monkeys: 50–90 ms, e.g., Godlove et al. 2011; Middelbrooks and Schall 2014). The SST paradigm presented here is especially designed for large quadrupeds (Streudel 1990). This is in comparison with the conventional SST paradigm, which requires a simple sequence of limb movement. Simple sequences of limb movements are suitable for species that are able to sit on the hind legs and move the front legs independently, such as rodents or monkeys (e.g., Eagle et al. 2008; Godlove et al. 2011). The paradigm described, however, requires the stopping of a complex sequence of movements involving the whole body, which is why we would expect a lengthening of the SSRT. The SSRT was not affected by the sheep’s individual weights. Thus, the stopping ability of the sheep was not influenced by inertia produced through the locomotory nature of the task. Comparing the SSRT recorded in this study to other studies is, therefore, difficult because this novel method measures the time to stop a full body movement instead of a single limb movement. The advantage of this method is that it provides a direct as opposed to an indirect measure of the reaction time requiring fewer trials. This direct measurement (through whole body movement) may, therefore, be advantageous for other species and thus the study provides a platform upon which further studies can be undertaken.

The stopping performance variance between sheep was significantly larger at the beginning of the experiment than at the end. Together with the increase in the number of correct responses and the reduction in the SSRT values, this demonstrates a training effect. This significant training effect is especially interesting, as we needed significantly fewer trials than used in other studies (450 trials in the current study, versus 18,000 trials in macaques (Middelbrooks and Schall 2014), and, 1000 trials in rats (Eagle et al. 2007).

We found a trend of increased stopping performance for animals with longer SSRTs during Sessions 1–5 of the experiment. This provides a first indication that, with little or no training, slower animals perform better in the task than faster and possibly more impulsive animals. An explanation could be that slower animals simply have more time to react to an upcoming stimulus. In contrast, at the end of the experiment, the inter- and intra-individual variance is significantly reduced, and all sheep show similar performance in their stopping behaviour in terms of the percentage of successful stops and the SSRT, again showing a strong training effect.

In animal studies, it is important to maintain high levels of motivation, which is usually achieved through reward and punishment (Warden 1931). Eagle and Robbins (2003) successfully introduced a version of the SST for rodents in which the rats had to respond within a restricted time frame. The time limit was set to a 10–15% increase in the individual go-RT. If the rats did not respond within the time limit, they were held in darkness for several seconds. With this implementation, the animals maintained a fast reaction performance throughout the experiment. The SST paradigm that we are presenting here allows a similar manipulation, which can be realised in the ‘timeout’ period of each trial. In the current experiment, we used a prolonged timeout period of 15 s per trial (nearly 600% of the mean go-RT). The time limit was only reached on a small number of trials (1–3%). Despite the prolonged timeout period used in our set-up, nearly all sheep significantly shortened their SSRT over the course of the experiment. Using a shorter timeout period would probably lead to a reduction in the variance for the go-RTs and SSRT values.

The SST paradigm has been studied in rhesus and bonnet macaque monkeys (e.g., Godlove et al. 2011, Godlove and Schall 2016, Middelbrooks and Schall 2014), primarily to explore the neural signature of response inhibition (e.g., Emeric et al. 2008). Our study shows that sheep have the ability to stop an initiated action in response to an operant cue, in a way that is comparable to established paradigms in humans and monkeys measured using established paradigms. We therefore propose that sheep could be used to replace non-human primates when investigating the neural signature of response inhibition. Furthermore, this paradigm provides the opportunity to use the SST to investigate the efficacy of therapeutic drugs relevant for HD. Based on pharmacological studies conducted using the SST for rats, we plan to conduct pharmacological tests using the SST for sheep to investigate potential therapeutic agents.

This is the first time a SST paradigm has been used in sheep. Our results show that sheep are not only able to stop a response that has already been started, but also that they are able to revise the response that was first initiated. We describe a version of the SST paradigm, which allows capturing a direct measure of the SSRT using a back-mounted accelerometer and significantly reduces the number of trials required to obtain reliable results. This SST paradigm, therefore, adds to the repertoire of tests suitable for investigating cognitive dysfunction and its progression in sheep models of neurodegenerative diseases such as the transgenic HD sheep.
